# Dural arteriovenous fistula masquerading as pulsatile tinnitus: radiologic assessment and clinical implications

**DOI:** 10.1038/srep36601

**Published:** 2016-11-04

**Authors:** Yong-Hwi An, Sungjun Han, Minhyung Lee, Jihye Rhee, O-Ki Kwon, Gyojun Hwang, Cheolkyu Jung, Yun Jung Bae, Gwang Seok An, Kyogu Lee, Ja-Won Koo, Jae-Jin Song

**Affiliations:** 1 Department of Otorhinolaryngology-Head and Neck Surgery, Eulji University, Eulji Medical Center, Seoul, Korea; 2 Department of Otorhinolaryngology-Head and Neck Surgery, Seoul National University Bundang Hospital, Seongnam, Korea; 3 Department of Neurosurgery, Seoul National University Bundang Hospital, Seongnam, Korea; 4 Department of Radiology, Seoul National University Bundang Hospital, Seongnam, Korea; 5 Music and Audio Research Group, Graduate School of Convergence Science and Technology, Seoul National University, Seoul, Korea

## Abstract

Pulsatile tinnitus (PT) is often an initial presenting symptom of dural arteriovenous fistula (dAVF), but it may be overlooked or diagnosed late if not suspected on initial diagnostic work-up. Here, we assess anatomical features, treatment outcomes, and clinical implications of patients with PT due to dAVF. Of 220 patients who were diagnosed with dAVF between 2003 and 2014, 30 (13.6%) presented with only PT as their initial symptom. The transverse-sigmoid sinus (70.0%) was the most common site, followed by the hypoglossal canal (10.0%) and the middle cranial fossa (6.7%) on radiologic evaluation. Regarding venous drainage patterns, sinus or meningeal venous drainage pattern was the most common type (73.3%), followed by sinus drainage with a cortical venous reflux (26.7%). PT disappeared completely in 21 (80.8%) of 26 patients who underwent therapeutic intervention with transarterial embolization of the fistula, improved markedly in 3 (11.5%), and remained the same in 2 (7.7%). In conclusion, considering that PT may be the only initial symptom in more than 10% of dAVF, not only otolaryngologists but also neurologists and neurosurgeons should meticulously evaluate patients with PT. In most cases, PT originating from dAVF can be cured with transarterial embolization regardless of location and venous drainage pattern.

Tinnitus, defined as the phantom perception of sound in the absence of a corresponding external source, is a term used for many forms of the symptom with various characteristics and different causes. The classification of tinnitus into pulsatile or non-pulsatile based on the perceived quality of the sound is of help to clinicians, because heartbeat-synchronous pulsatile tinnitus (PT) is predominantly vascular in origin^1^,^2^,^3^,^4^. PT usually results from vibrations of turbulent blood flow in vessels inside or near the middle ear^5^. Although PT is uncommon and represents less than 10% of all tinnitus^6^, it is important to recognize this category of tinnitus because PT is surgically curable when the causative vascular abnormalities are determined and resolved. In the clinical setting for patients with tinnitus, the management of PT is somewhat challenging due to its infrequency and lack of standardized diagnostic and therapeutic protocols.

Of known underlying diseases, intracranial dural arteriovenous fistula (dAVF) is one of the most common causes of arterial pulse synchronous PT^7^,^8^. dAVF indicates an abnormal direct connection between dural arteries and dural veins or a venous sinus, accounting for 10–15% of intracranial arteriovenous malformations^9^,^10^. Patients with dAVF can be asymptomatic or can experience symptoms, ranging from mild PT to fatal intracranial hemorrhage, depending on the anatomical location and venous drainage pattern^11^. PT is often the sole initial symptom of dAVF, but a high index of suspicion and an appropriate evaluation are essential to avoid misdiagnosis and potentially catastrophic consequences. Cross-sectional images using contrast-enhanced temporal bone computed tomography and brain magnetic resonance imaging with angiography (MRI/A) give useful information for the diagnosis of dAVF, but for a complete characterization and classification of dAVF, classic angiography should be performed^12^.

dAVFs have been managed with conservative treatment, neurosurgical resection, venous clipping, endovascular embolization, radiation therapy, and combinations of these methods^11^,^12^. Although surgery still plays an important role in some complex cases, most patients with dAVF can be treated successfully with transarterial or transvenous selective embolization. Considering that most dAVFs are curable with presently available treatment modalities, accurate diagnosis of dAVF presenting with only PT by performing a meticulous physical examination and choosing appropriate neuroimaging modalities is essential.

Many researchers have documented radiological findings and treatment outcomes of dAVF from a neurosurgical viewpoint, but there are few studies on practical guidance for patients presenting with only PT obviously due to intracranial dAVF. Thus, we sought to evaluate retrospectively the clinical features, anatomical details, and treatment results in dAVF patients presenting with only PT. We also investigated the effects of potential influencing factors on the treatment outcomes.

## Methods

### Subjects

We conducted a retrospective review of the medical records, brain MRI/A, and transfemoral cerebral angiography (TFCA) findings of 220 patients who were diagnosed with dAVF at Seoul National University Bundang Hospital between January 2003 and December 2014. Of them, a total of 30 patients (13.6%) visited the department of otolaryngology or neurosurgery with only PT as their initial symptom. Patient ages ranged from 27 to 80 years (mean, 52.8 ± 11.7 years). Of the 30 patients, 8 were men and 22 were women; 26 underwent therapeutic intervention with transarterial embolization of the fistula during TFCA. Detailed patient characteristics are summarized in Table 1. The study was approved by the institutional review board of the Clinical Research Institute at the hospital (IRB#: B-1601-332-103) and informed consent was obtained from all subjects. The study procedures were carried out in accordance with the relevant guidelines and regulations.

### Physical examination, audiological evaluation, and transcanal sound recording

On physical examination, the head-and-neck area was fully examined including changes in PT on digital compression of the ipsilateral internal jugular vein and head rotation to the ipsi- and contralateral side, and auscultation to locate any possible source of the PT. Pure tone audiometry was conducted in nine patients who visited the department of otolaryngology at the initial visit. The pure tone average was determined at 0.5, 1, 2, and 4 kHz. Hearing asymmetry of low-tone was defined as (1) a low-tone threshold discrepancy of 10 decibel (dB) or more on at least two consecutive frequencies (0.25 and 0.5 kHz) or a discrepancy of more than 20 dB on at least one frequency (0.25 or 0.5 kHz), as determined by pure-tone audiograms, and (2) the pure tone thresholds at low frequencies in the tinnitus-affected ear greater than that of the unaffected ear^13^. In the last four patients of the current case series, as a recording system became available, the PT acoustic characteristics were analyzed via transcanal tinnitus sound recording, using an inserted microphone (RODE microphones, Sydney, Australia), real-time recording, using the Cubase 5.0 software (Steinberg Media Technologies GmbH, Hamburg, Germany), and data analysis with the aid of MATLAB R2013a (MathWorks, Natick, MA, USA). The recording and analysis methods are further described in recent articles^14^,^15^.

### Diagnostic evaluation and endovascular interventions

**Figure 1:**
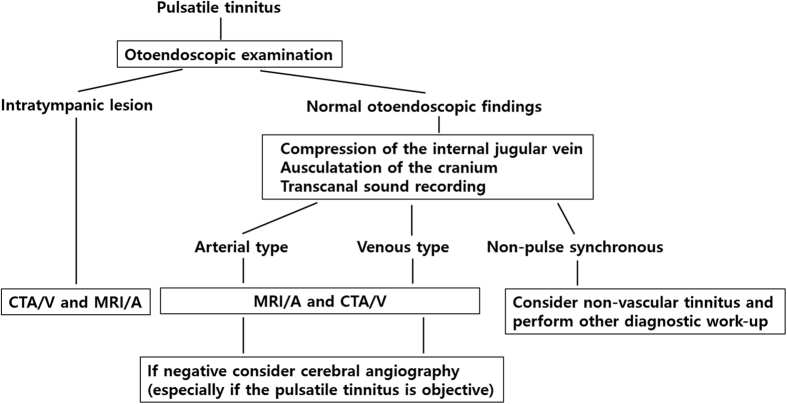
Diagnostic algorithm for pulsatile tinnitus. IJV = internal jugular vein, CTA/V = combined CT angiography and venography.

All patients underwent directed evaluation in terms of the side of the tinnitus, its duration, and other otologic symptoms. When the patient initially complained of heartbeat-synchronous PT, the head-and-neck area was fully examined, including changes in PT upon digital compression of the ipsilateral internal jugular vein and auscultation to locate any possible source of PT. If dAVF was highly suspected due to the patient’s history and physical findings, brain MRI/A was checked to detect intra- or extratemporal vascular etiologies including dAVF. Not to miss the diagnosis with dAVF in cases of PT, a solid diagnostic algorithm for patients with PT is necessary. Figure 1 summarizes the suggested diagnostic algorithm for a patient with pulsatile tinnitus. If dAVF was relevant to PT, TFCA was recommended to find definite feeding arteries and draining veins and to classify the lesion by anatomical similarities on the basis of the Borden classification^16^. After confirming the exact vascular anatomy of dAVF, endovascular embolization therapy was preferred as the primary treatment modality. N-butyl 2-cyano-acrylate was used as an embolic glue in most cases, and platinum coils were used in some cases. Embolization was terminated by confirming that the liquid adhesive embolic agent reached the proximal draining veins via the fistula nidus.

If a patient’s symptom completely disappeared, we checked follow-up brain MRI/A 6 months after the embolization, and then checked brain MRI/A yearly for 2 to 3 years. If there was any residual symptom after the embolization, surgical disconnection or radiosurgery were considered in cases of definite residual target lesion.

### Outcome Measures

Subjective improvements in PT were measured 6 months after embolization by determining the changes in global symptoms and were categorized into the following five types: (1) completely disappeared and ‘cured,’ (2) markedly improved, (3) slightly improved, (4) unchanged, and (5) worsened. For 6 patients whose post-treatment follow-up duration was less than 6 months, telephone interview regarding subjective symptom improvements was performed. The treatment outcomes were compared between subgroups divided by the anatomical location and venous drainage pattern of the dAVF according to the TFCA findings. We examined the associations of the cure- and improvement rates with clinical parameters, such as age, gender, duration of symptoms, the side of PT, intracranial location of the lesion, and drainage pattern, according to the Borden classification.

## Results

### Patient Characteristics

Of 30 patients with PT, 29 had unilateral tinnitus and one had bilateral tinnitus (Table 1). In patients with unilateral tinnitus, the right ear was affected in 9 patients and the left ear in 20. In all patients with unilateral tinnitus, the sides of intracranial dAVF corresponded with the direction of PT. In the only patient with bilateral tinnitus, the dAVF was located at the confluence of sinuses (i.e. torcula), the midline connecting point of the superior sagittal sinus, straight sinus, and occipital sinus. This patient and another three with dAVF at each jugular bulb and transverse-sigmoid sinus were observed with no angiographic management. The mean duration of tinnitus perception was 9.8 ± 22.3 (range, 1–120) months. The average follow-up duration was 24.2 ± 28.4 (range, 5–132) months.

### Audiometric profiles and spectro-temporal analysis results

Among nine patients who underwent pure tone audiometry, one subject with dAVF at the midline torcula and bilateral PT was excluded from the audiometric profile analysis. Of eight patients included in the audiometric profile analysis, four showed audiological asymmetry: one patient showed a low-tone threshold discrepancy of 10 dB or more on more than two consecutive frequencies and three patients showed a discrepancy of more than 20 dB on at least one frequency on the ipsi-lesional side.

**Figure 2:**
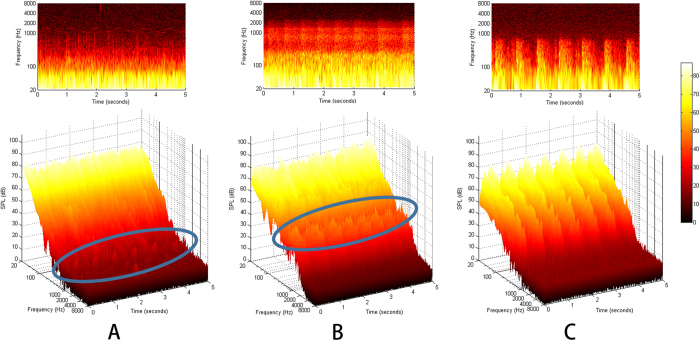
Three patients’ recorded signals represented by two-dimensional spectrograms and three-dimensional waterfall diagrams.

Of four patients who underwent transcanal sound recording, three showed definite periodic, pulse-synchronous acoustic features (Fig. 2). Two of those patients displayed unique pulsatile bumps at ~1500 Hz that reached an audible SPL (see pulsatile bumps in solid line ellipse in Fig. 2A,B), and the other exhibited large peak amplitudes and a periodic structure with a broadband nature (Fig. 2C).

### Radiologic Findings

**Figure 3:**
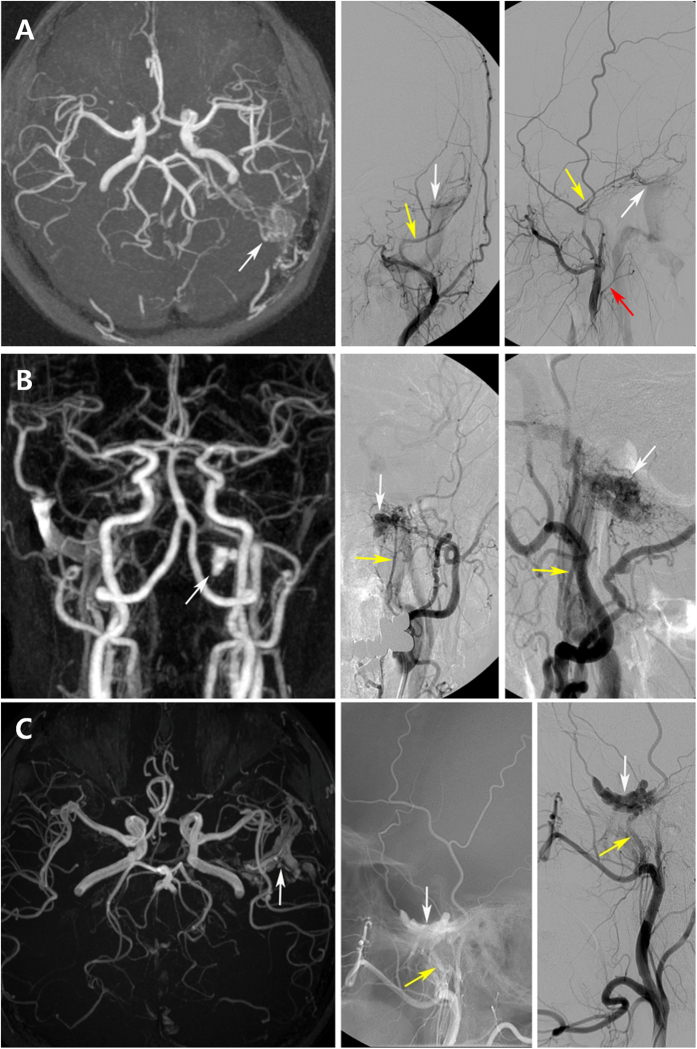
Representative brain MRI images and TFCA findings of (**A**) transverse-sigmoid sinus dAVF, (**B**) hypoglossal canal dAVF, and (**C**) middle cranial fossa dAVF. (**A**) Left external carotid artery angiogram in anteroposterior and lateral views shows a dAVF with feeders from left middle meningeal artery (yellow arrows) and left occipital artery (red arrow), draining into left sigmoid sinus (white arrows). (**B**) Left external carotid artery angiogram in anteroposterior and lateral views shows a dAVF with feeders from left ascending pharyngeal artery (yellow arrows), draining into left internal jugular vein (white arrows at venous sac). (**C**) Unsubtracted and subtracted left external carotid artery angiograms in lateral view show a dAVF in left middle cranial fossa with feeders from left middle meningeal artery (yellow arrows), draining into left inferior petrosal sinus (white arrows at venous sac).

The transverse-sigmoid sinus (21 cases, 70.0%) was the most common site of dAVF triggering PT, followed by the hypoglossal canal (3, 10.0%) and the middle cranial fossa (2, 6.7%; Fig. 3, Table 2). The venous drainage directly into the dural venous sinus or meningeal vein (Borden type I; 22 cases, 73.3%) pattern was more common than sinus drainage with cortical venous reflux (Borden type II; 8 cases, 26.7%; Table 3). Among the eight dAVFs with a Borden type II venous drainage pattern, seven were located at the transverse-sigmoid sinus and one at the confluence of sinuses (torcula). No patient in our series showed venous drainage directly into subarachnoid veins with cortical venous reflux only (Borden type III) on TFCA.

### Treatment Outcomes

**Figure 4:**
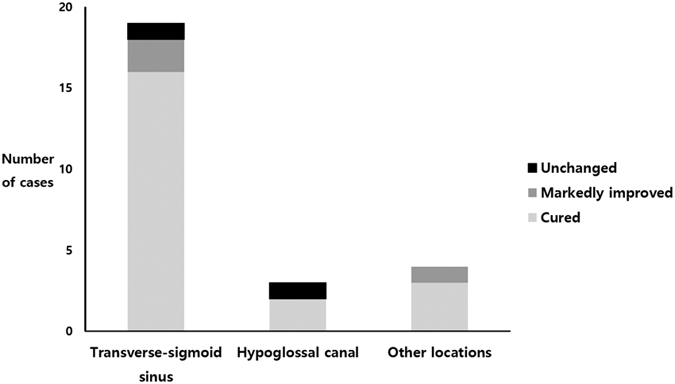
Comparison of treatment outcomes according to the anatomical location of the dAVF in patients who received transarterial embolization (N = 26).

Of the 26 patients (M:F = 7:19) who were managed by endovascular embolization of the dAVF, PT disappeared completely in 21 (80.8%), was abated substantially in 3 (11.5%), and remained the same in 2 (7.7%), resulting in a subjective symptom improvement rate of 92.3%. No patient reported slightly improved or worsened symptom after definite treatment of dAVF. Four patients who were followed up without any surgical or interventional treatment showed no change in PT during the follow-up period. Of 21 patients with dAVF at the transverse-sigmoid sinus, 19 underwent endovascular embolization; of these, the symptom disappeared in 16 (84.2%) patients, was markedly improved in 2, and was unchanged in 1. Of three patients with dAVF at the hypoglossal canal, PT disappeared in two (66.7%) and the other showed unchanged symptom (Fig. 4). Of 22 patients with a Borden type I drainage pattern, 19 were treated with endovascular embolization; of these, PT was cured in 14 (73.7%), improved substantially in 3, and remained the same in 2. Seven of eight patients with Borden type II drainage patterns underwent endovascular embolization and all presented with complete resolution of PT. One Borden type II patient was not treated by transarterial or transvenous embolization due to the patient’s poor general medical condition and small amount of cortical reflux. No major complications occurred after endovascular treatment.

## Discussion

A few previous studies have described ear complaints and demonstrated therapeutic outcomes of endovascular intervention for PT due to dAVF as well as other vascular causes^8^,^17^,^18^,^19^. However, in this study, we focused on data from our large case series of dAVF presenting with only PT to provide our colleague clinicians helpful information on the diagnosis and treatment of this challenging disease entity. To sum up, PT was the only initial symptom in more than 10% of dAVF, and the transverse-sigmoid sinus was the most common site of dAVF triggering PT. PT improved in 92.3% of cases after endovascular embolization, and there were no significant differences in the cure rate according to anatomical location or drainage pattern.

The transverse-sigmoid sinus (70.0%) was the most common site of dAVF that presented solely with PT, followed by the hypoglossal canal (10.0%) and the middle cranial fossa (6.7%). Anatomical proximity of the lesion to the inner ear results in relatively more frequent presentation with PT in dAVFs originating from the transverse and sigmoid sinus compared to dAVFs from other vascular structures^11^,^12^,^18^. In a previous study, PT was the chief complaint in 90% of patients with transverse/sigmoid sinus dAVF^16^. Although the most common symptoms of transverse/sigmoid sinus dAVF are benign ones, such as PT or mild to moderate headache, lesions in this region are thought to be more closely associated with hemorrhagic and aggressive neurological symptoms than cavernous sinus dural AVFs^11^. In our series, the proportion of retrograde sinus drainage with cortical venous reflux (Borden type II) in transverse/sigmoid dAVFs (33.3%) was higher than that of dAVFs at other locations (11.1%). Considering that the Borden type II drainage pattern is more closely related to fatal complications, clinical suspicion and proper diagnosis of transverse/sigmoid dAVF is particularly important if it presents with only PT. Three patients presenting with only PT had dAVF at the hypoglossal canal. According to a recent systematic literature review on hypoglossal canal dAVF, ~75% of such patients present with PT, and PT is often the only symptom in hypoglossal canal dAVFs with solely anterograde venous drainage^20^. Our case series is consistent with those findings in that all three patients with hypoglossal canal dAVF presented solely with PT and all of them showed Borden type I drainage patterns on TFCA. Considering anatomical proximity, PT perception in patients with hypoglossal canal dAVFs may also be attributable to direct transmission of the venous bruit to the inner ear structure through the temporal bone. In addition, in the other seven patients, dAVFs were identified in areas such as the middle cranial fossa, cavernous sinus, occipital area, jugular bulb, and confluence of sinuses (torcula). Because more than 10% of dAVFs presented with only PT in our series and those intracranial dAVFs can be located anywhere near the inner ear structure, when a patient visits an outpatient clinic with a sole complaint of PT, the possibility of dAVFs should always be considered, inasmuch as they may visit otolaryngologists, neurosurgeons, or neurologists.

Of 26 patients who underwent endovascular embolization, 24 (92.3%) reported improvement of their PT; of these, 21 (80.8%) achieved complete resolution of PT and the symptoms abated greatly in the other 3 (11.5%). This cure rate is comparable to that of several case series with PT^17^,^18^,^21^, and to that of dAVF with other symptoms^11^,^12^. During the past two decades, endovascular management through transarterial, transvenous, or combined approaches has become the first-line treatment for dAVFs. While high-grade lesions with cortical venous reflux should be treated as soon as possible to avoid the risks of hemorrhage, low-grade dAVFs with severe debilitating PT imposing poor quality of life may also be candidates for prompt endovascular repair. In addition, if PT is the only presenting symptom in patients with dAVF, prompt management by endovascular embolization can prevent further neurological and neurosurgical complications. It is notable that there were no significant differences in treatment outcomes among the patients with regard to various anatomical features, including intracranial locations and drainage patterns. If total occlusion of the shunt is not achievable or considered too dangerous, selective disconnection of cortical venous reflux is recommended to prevent neurological morbidity^22^. Two patients had persistent PT after TFCA because numerous feeders of dAVF precluded complete embolization: both had Borden type I drainage patterns, one was located at the transverse-sigmoid sinus and the other was at the cavernous sinus. Nevertheless, endovascular embolization is recommendable as the initial treatment of choice for these benign types of dAVFs because TFCA carries a relatively low rate of morbidity, and the stable natural history of these dAVFs does not justify the risk of sinus sacrifice.

General approaches for the management of dAVFs include conservative treatment, endovascular intervention, surgery, and radiosurgery. Due to the recent efficacy of endovascular therapy, microsurgical obliteration is often reserved for cases in which endovascular embolization has failed or is not feasible^11^,^12^,^23^. Surgical disconnection of dAVFs has shown excellent results as well, and cases involving dAVFs of the floor of the anterior cranial fossa and the superior sagittal sinus can often be treated more easily and safely with surgical approaches^12^,^24^. The cure rate of surgical elimination of the dAVF has been reported to be nearly 100%, but the risk of transient and permanent morbidity remains up to 10%^12^,^25^. Studies on stereotactic radiosurgery have reported relatively good outcomes, with complete occlusion rate of 44–87% without major complications^11^,^12^. Although we preferred embolization via a transarterial method, the optimal way of endovascular treatment remains controversial. The rates of complete ablation by transvenous embolization have been reported to be 71–100%^11^,^12^. Because choice of transarterial, transvenous, and combined approaches depends mainly on dAVF architecture, pattern of venous drainage, location and clinical presentation, we agreed that individualized endovascular treatment result in a higher degree of cure rate with a lower complication rate. However, we could reasonably managed vascular PT in patients with dAVF by transarterial route with total symptomatic resolution of 80.8% in this study population.

Previous literature on vascular PT has indicated that ipsi-lesional hearing loss is observed in some patients with PT and this is probably due to the masking effect of PT sound^3^,^26^,^27^. In the current case series, also, 50% of the patients evaluated by pure tone audiometry exhibited audiometric asymmetry. They showed either ipsi-lesional higher thresholds at one mid-frequency range or overall higher thresholds from low to middle frequencies. These audiometric results are also consistent with the preliminary data of four patients who were evaluated by spectro-temporal analysis of the recorded transcanal signal. Their data exhibited either unique pulsatile bumps at the mid-frequency range that reached an audible SPL or large peak amplitudes and a periodic structure with a broadband nature (Fig. 2). These results are presumably due to the nature of blood flow through the fistula tract. That is, when the fistulous tract is narrow, it could be surmised that there is a relatively small volume of blood flow through the narrow fistulous tract and this may generate PT with relatively higher frequency but small amplitude. Then, if the fistulous tract becomes wider, a larger amount of blood flow may generate PT with a larger amplitude and a broadband nature. However, this should be confirmed by future studies on a larger number of patients with dAVF.

To the best of our knowledge, this is the first report on the clinical characteristics and treatment results for dAVF patients presenting with only PT as their initial symptom. However, our case series was limited in several aspects. First, the number of subjects was relatively small and the mean follow-up period was relatively short; thus, we could not draw conclusions about the outcomes and influencing factors of Borden type III, in-depth audiological profiles, any correlation with the location and extent of dAVF, or long-term follow-up results. Further clinical experience with a larger patient group is required to further evaluate clinical characteristics and treatment results. Moreover, we could only indirectly assess symptom improvement using subjective scales because we cannot yet objectively compare pre- and post-treatment symptoms. In this regard, further work on objective measurements of PT is warranted.

In conclusion, given that PT can be the only initial symptom in more than 10% of dAVF, not only otolaryngologists but also neurologists and neurosurgeons should meticulously evaluate patients with PT to rule out the possibility of dAVF via a thorough history taking, physical examination, and audiological and psychoacoustic evaluations. When suspected, brain MRI/A and TFCA should be performed to diagnose and manage dAVF. In most cases, PT originating from dAVF can be cured by transarterial embolization regardless of the location and venous drainage pattern.

## Additional Information

**How to cite this article**: An, Y.-H. *et al*. Dural arteriovenous fistula masquerading as pulsatile tinnitus: radiologic assessment and clinical implications. *Sci. Rep.* **6**, 36601; doi: 10.1038/srep36601 (2016).

**Publisher’s note:** Springer Nature remains neutral with regard to jurisdictional claims in published maps and institutional affiliations.

## Figures and Tables

**Table 1:** Demographic and clinical characteristics of the 26 patients with dural arteriovenous fistula presenting only with pulsatile tinnitus.

Case No.	Age (years)	Sex	Side	Symptom Duration (months)	Location of Fistula	Borden type	PT	F/U period (months)
1	67	F	R	36	Occipital area	I	C	35
2	41	M	L	4	Transverse-sigmoid	I	C	8
3	46	M	L	1	Middle cranial fossa	I	C	58
4	60	F	B	6	Torcula	II	U*	8
5	38	M	L	3	Transverse-sigmoid	I	U*	6
6	60	M	R	30	Jugular bulb	I	U*	15
7	62	F	L	8	Transverse-sigmoid	I	C	54
8	60	F	L	12	Transverse-sigmoid	I	C	5
9	50	F	L	3	Transverse- sigmoid	II	C	31
10	53	F	L	3	Transverse- sigmoid	I	I	54
11	80	F	L	3	Hypoglossal canal	I	C	5
12	50	F	L	2	Transverse-sigmoid	I	C	21
13	50	F	L	3	Hypoglossal canal	I	S	8
14	49	F	L	5	Transverse-sigmoid	I	I	37
15	41	F	L	1	Transverse-sigmoid	I	C	33
16	64	F	L	3	Transverse-sigmoid	II	C	82
17	37	F	L	6	Transverse-sigmoid	I	C	19
18	50	F	L	3	Transverse-sigmoid	II	C	12
19	50	F	L	9	Transverse-sigmoid	II	C	5
20	69	M	L	120	Transverse-sigmoid	II	C	28
21	56	F	R	4	Transverse-sigmoid	I	U	17
22	27	F	R	1	Middle cranial fossa	I	C	9
23	66	F	R	10	Transverse-sigmoid	II	C	132
24	50	F	R	1	Cavernous sinus	I	I	9
25	52	M	R	1	Hypoglossal canal	I	C	5
26	40	M	R	3	Transverse-sigmoid	II	C	8
27	42	M	L	2	Transverse-sigmoid	I	C	5
28	49	F	L	2	Transverse-sigmoid	I	C	5
29	72	F	L	5	Transverse-sigmoid	I	C	6
30	53	F	R	3	Transverse-sigmoid	I	U*	5

M, male; F, female; R, right; L, left; B, bilateral; PT, pulsatile tinnitus; C, cured; I, improved; U; unchanged; *these cases were observed with no angiographic management.

**Table 2:** Anatomical location of intracranial dural arteriovenous fistulas (N = 30).

Area	Number of patients	Percentage
Transverse-sigmoid sinus	21	70.0%
Hypoglossal canal	3	10.0%
Middle cranial fossa	2	6.7%
Cavernous sinus	1	3.3%
Occipital area	1	3.3%
Jugular bulb	1	3.3%
Confluence of sinuses (torcula)	1	3.3%

**Table 3:** Borden classification of dural arteriovenous fistula^25^.

Type	Venous Drainage	N
I	Direct drainage of meningeal arteries to a meningeal vein or dural venous sinus with normal antegrade flow	22 (73.3%)
II	Shunts between the meningeal arteries and dural sinus, with cortical venous reflux into the subarachnoid veins	8 (26.7%)
III	Venous drainage directly into subarachnoid veins (i.e., cortical venous reflux only)	0
